# Gnocis: An integrated system for interactive and reproducible analysis and modelling of *cis*-regulatory elements in Python 3

**DOI:** 10.1371/journal.pone.0274338

**Published:** 2022-09-09

**Authors:** Bjørn André Bredesen-Aa, Marc Rehmsmeier

**Affiliations:** 1 Computational Biology Unit, Department of Informatics, University of Bergen, Bergen, Norway; 2 Department of Biology, Humboldt-Universität zu Berlin, Berlin, Germany; Yeshiva University Albert Einstein College of Medicine, UNITED STATES

## Abstract

Gene expression is regulated through *cis*-regulatory elements (CREs), among which are promoters, enhancers, Polycomb/Trithorax Response Elements (PREs), silencers and insulators. Computational prediction of CREs can be achieved using a variety of statistical and machine learning methods combined with different feature space formulations. Although Python packages for DNA sequence feature sets and for machine learning are available, no existing package facilitates the combination of DNA sequence feature sets with machine learning methods for the genome-wide prediction of candidate CREs. We here present Gnocis, a Python package that streamlines the analysis and the modelling of CRE sequences by providing extensible APIs and implementing the glue required for combining feature sets and models for genome-wide prediction. Gnocis implements a variety of base feature sets, including motif pair occurrence frequencies and the k-spectrum mismatch kernel. It integrates with Scikit-learn and TensorFlow for state-of-the-art machine learning. Gnocis additionally implements a broad suite of tools for the handling and preparation of sequence, region and curve data, which can be useful for general DNA bioinformatics in Python. We also present Deep-MOCCA, a neural network architecture inspired by SVM-MOCCA that achieves moderate to high generalization without prior motif knowledge. To demonstrate the use of Gnocis, we applied multiple machine learning methods to the modelling of *D. melanogaster* PREs, including a Convolutional Neural Network (CNN), making this the first study to model PREs with CNNs. The models are readily adapted to new CRE modelling problems and to other organisms. In order to produce a high-performance, compiled package for Python 3, we implemented Gnocis in Cython. Gnocis can be installed using the PyPI package manager by running ‘pip install gnocis’. The source code is available on GitHub, at https://github.com/bjornbredesen/gnocis.

## Introduction

Gene expression is regulated through *cis*-regulatory elements (CREs) [1]. Multiple classes of CREs have been identified, with functions ranging from directly stimulating target gene activity [2] over maintaining epigenetic memory [3] to delimiting the effects of other CREs [4]. CREs are typically enriched in a variety of protein binding sites which can be characterized as sequence motifs [5].

Advances in experimental methods have given rise to a growing body of genome-wide experimental data from a multitude of organisms, capturing binding patterns of DNA- and chromatin-binding proteins, histone tail modifications, chromatin conformation, and DNA accessibility [6]. Given a set of CREs, analyses of the underlying sequences can shed light on the defining sequence criteria and enable the training of predictive models. Knowledge of the defining sequence criteria can yield new insights about the function of the CRE class under investigation, and predictive models can yield predictions beyond the confines of available experimental data.

We previously observed improved generalization when training models with genome-wide experimental data for Polycomb/Trithorax Response Elements (PREs) [7], a CRE class that maintains epigenetic memory [8]. Given sets of relevant experimental data for a CRE class of interest, several steps are necessary in order to produce candidate CRE predictions, including data preparation, model specification, comparison with alternative models and genome-wide prediction. Models of CRE sequences can be specified in a number of ways, for example by combining a feature set with a machine learning method, such as Support Vector Machines (SVMs) [9] or Random Forests (RFs) [10]. Feature spaces for CRE sequence models can also be defined in numerous ways, including singular and paired motif occurrence frequencies [3] and k-spectra—the set of occurrence frequencies of all motifs of length k—with or without mismatches allowed [11]. Alternatively, Convolutional Neural Networks (CNNs) can be used to learn predictive features directly from data without the need for a predefined feature set and have yielded notable success for complex recognition tasks such as in the area of image classification [12].

In order to decide what models yield the best generalization, unbiased comparison should be employed, for example using cross-validation on the same training and test data for all models. When data is highly imbalanced, as is typically the case with CREs versus non-CREs, the Precision/Recall curve reflects expected generalization in light of the imbalance [13]. The use of Jupyter Notebooks [14] enables interactive and reproducible workflows in Python, with integrated visualization.

Powerful machine learning packages are available for Python, such as Scikit-learn [15] for classical machine learning and TensorFlow [16] for neural networks. Packages also exist for Python that enable the specification of motifs and the search for their occurrences [17] and for k-spectrum feature sets [18–20]. These contributions notwithstanding, a package that provides base functionality for combining machine learning methods with DNA sequence feature sets has been absent, leaving the end-user to implement this functionality on his or her own. Such functionality includes the bridging of outputs of feature set modules and inputs of machine learning models, the scoring of sequences using sliding windows, prediction threshold calibration and genome-wide prediction. Additionally, such a package could simplify the comparison of alternative models and feature sets. It could also implement a variety of optimizations to reduce run-time cost for the end-user, for example through parallel model application and efficient data handling.

We here present Gnocis (read no-cis), a Python package that streamlines the modelling of CRE sequences. Gnocis facilitates interactive and reproducible CRE sequence analysis and machine learning by providing a broad suite of tools for data preparation and analysis, a flexible vocabulary for the specification of feature sets, flexible and extensible APIs for feature set and model specification, and base functionality for the combination of machine learning methods and feature sets and for the application of models. The broad suite of data preparation and handling functionality implemented in Gnocis also makes our package useful for more general DNA bioinformatics in Python. Gnocis facilitates interactive use through integration with IPython [21] and provides interoperability with existing packages through integration with NumPy [22] and Pandas [23, 24]. In order to facilitate model comparison, Gnocis provides a cross-validation engine that supports imbalanced, multi-class data. Gnocis is open source and extensible and can be installed via the PyPI package manager.

## Implementation

### A rich vocabulary for the preparation and interactive analysis of genomic data

Multiple types of data are relevant for CRE machine learning, including DNA sequences, genomic region coordinates and genome-wide factor binding profiles. For example, we previously used genomic coordinates of experimentally determined clusters of Polycomb/Trithorax binding data to train sequence models of PREs [7]. Multiple formats have been formulated for DNA sequences (e.g. FASTA and 2bit) and genomic regions (e.g. GFF and BED). A variety of operations on data are useful for the preparation of training data, including the clustering of experimentally determined protein binding data and the extraction of the underlying DNA sequences. Although packages exist for the handling of DNA sequences [17] and genomic regions [25], they each support only subsets of relevant file formats, and interoperability is limited, requiring code to bridge them. The Pandas [23, 24] package provides a broad suite of intuitive tools for preparing and handling tabular data in Python and has achieved high popularity in the data science community.

Inspired by the success of Pandas, we saw that there was an opportunity for improving on how data can be handled and prepared in Python for CRE machine learning by providing a broad suite of tools for handling multiple types of data, with interoperability and support for established file formats. To facilitate the preparation and handling of sequence data, we implemented classes for DNA sequences, with support for loading both FASTA and 2-bit format files. To optimize memory efficiency, we implemented support for streaming sequences in chunks from disk, including the streaming of sliding windows with a desired length and step size. This avoids the need for loading large sequences to memory. Generative sequence models can be useful for defining negative training data, and we accordingly implemented an i.i.d. sequence generator and an nth-order Markov chain. To facilitate the preparation of genome-wide region data, we implemented classes for regions and for sets of regions, and we implemented a broad selection of transformation operations, including intersection, merging and exclusion and the acquisition of overlaps and non-overlaps. Gnocis supports the loading of regions in General Feature Format (GFF), Browser Extensible Data format (BED) and as coordinate lists. We also implemented the extraction of underlying sequences based on sets of regions and source sequences or a genome. Genome-wide curves are useful for representing experimentally determined genome-wide binding of factors and for scores made by predictive models. We implemented a class for handling curves, with support for the saving and loading of Wiggle format files, and with functionality for deriving a set of regions by thresholding. With the aim of high expressiveness with minimal verbosity, we implemented operations on data as transformations that can be chained, with short and intuitive naming. For run-time efficiency, we implemented the sequence and region data handling in Cython. The data preparation facilities of Gnocis are listed in Table 1.

**Table 1:** Core data preparation features.

**Sequence file operations**	FASTA (loading/saving)
	2bit (loading)
	Streaming from disk
	Sliding window extraction
**Region file operations**	Coordinate lists (loading/saving)
	General Feature Format (GFF) (loading/saving)
	Browser Extensible Data (BED) (loading/saving)
	G-zipped GFF (loading)
	G-zipped BED (loading)
**Curve file operations**	Wiggle (loading/saving)
	G-zipped Wiggle (loading)
	Thresholding
**Region set operations**	Merge
	Intersect
	Exclude
	Get overlapping
	Get non-overlapping
	Resize
	Randomly recentre
	Extract underlying sequences
**Genome operations**	Genomic sequences via sequence file operations
	Loading of annotation (Ensembl General Transfer Format, GTF)
**Biomarker set operations**	Define biomarker set based on sets of experimental signals
	Extract highly biomarker-enriched (HBME) genomic regions
	Extract lowly biomarker-enriched (LBME) genomic regions
**Generative sequence models**	Training of i.i.d. sequence model and generation of sequences
	Training of nth-order sequence model and generation of sequences
**Visualization**	Plotting of genomic regions and curves with Matplotlib [26]
	Plotting barplots of region overlap statistics with Matplotlib

Gnocis supports standard file formats for regions, curves and sequences, and implements a wide selection of operations in order to facilitate data preparation and handling.

### An expressive language for the specification and application of DNA sequence feature sets

In order to train machine learning models on DNA sequences, a mapping must be established from the input sequences to numerical vectors, where the mapping is commonly referred to as a feature set. Among the feature sets that have been successfully employed for CRE machine learning are motif occurrence frequencies [3, 7, 27] and k-spectra [28, 29]. A variety of packages useful for DNA sequence analysis in Python have been published, including packages that implement motif occurrence search [17] and DNA sequence feature sets such as k-spectra [18–20]. However, a Python package for generating feature sets based on known motifs, such as motif pair occurrence frequencies, is absent. Furthermore, existing packages do not provide or employ a general and extensible API for DNA sequence features with base functionality such as sequence window application.

We noticed the potential for a general feature set API to facilitate powerful and flexible feature set specification, combination and filtering of features, and efficient feature extraction for DNA sequence analysis and machine learning. We also noticed that certain feature sets can be most efficiently extracted in bulk, including motif pair occurrence frequencies and k-spectra. In order to exploit this efficiency, we implemented feature sets in Gnocis as directed, acyclic graphs, henceforth referred to as feature networks. The input nodes of a feature network are base feature sets (such as k-spectra), and subsequent nodes are transformations. Transformations can be chained, facilitating short and flexible feature network specification. Feature network nodes can be trained recursively, enabling feature scaling and model training. Base features and transformations implemented in Gnocis are listed in Table 2.

**Table 2:** Sequence feature analysis.

**Motifs**	IUPAC nucleotide code motifs
	Position Weight Matrices (PWMs)
	Transformation of motif sets into feature sets
**Base feature sets**	Motif occurrence frequencies
	Motif pair occurrence frequencies
	k-spectrum kernel
	k-spectrum mismatch kernel
**Feature set transformations**	Combination of feature sets
	Filtering of feature sets
	Feature pairing by product
	Scaling
**Feature tables**	Construction of feature value tables
	Output of summary statistics
	Output of differential summary statistics
	Conversion to NumPy [22] array
	Conversion to Pandas [23] data frame

Gnocis provides a flexible framework for the specification of sequence feature sets and integrates with NumPy [22] and Pandas [23] for analyses with external packages.

For ease of use, we implemented base functionality for extracting features from sequences, including the extraction of features from sliding windows of sequences streamed from disk. To facilitate interoperability with existing analytical tools, we implemented the output of features to NumPy [22] arrays and Pandas data frames [23]. We implemented the feature network system in Cython, which resulted in a compiled module with efficient feature extraction.

### A flexible and extensible modelling API provides the glue required for efficiently combining sequence feature sets and machine learning methods and to apply them for prediction

A variety of machine learning methods have been developed that can be combined with arbitrary numerical feature sets, including Support Vector Machines [9] and Random Forests [10]. In order to successfully apply these methods for the modelling and genome-wide prediction of CREs, bridging between the feature sets and the machine learning methods is required, and also implementations of sequence scoring and genome-wide application. Models not based on feature sets, such as DNA sequence Convolutional Neural Networks, also require this logic for performing genome-wide prediction. Genome-wide prediction can output scores for sliding windows, and thresholding can yield discrete predictions of candidate CREs. To our knowledge, no prior Python package has been published that implements this base functionality, leaving the prospective CRE modeller to implement this logic on his or her own.

We were interested in the potential ease of use and experimentation that a general DNA sequence modelling API could enable, and we implemented a modelling API with logic for sequence scoring, prediction threshold calibration and genome-wide prediction. Model application can be performed in parallel, and we implemented the optional use of multiprocessing. We further noticed that a flexible and compact model specification could be achieved by extending the feature network system. We added the transformation of feature sets into sequence models, given a base model as an argument. The recursive training of feature networks, including scaling and modelling methods, enables the re-training of models on new data, for example for cross-validation. Multi-class model training in Gnocis is achieved by assigning labels to sequences. We implemented a log-odds base model, and we created wrappers for Scikit-learn [15] implementations of Support Vector Machines (SVMs) and Random Forests. For SVMs, we additionally implemented GPU-based kernel application with CuPy [30]. We also implemented DNA sequence Convolutional Neural Networks and general Neural Networks via Keras [31] by integrating with TensorFlow [16]. We list features of the Gnocis modelling API in Table 3.

**Table 3:** Models.

**Base models**	Unweighted sum
	Log-odds
	Support Vector Machine via Scikit-learn [15]
	Support Vector Machine with GPU application via Scikit-learn [15] and CuPy [30]
	Random Forest via Scikit-learn [15]
**Sequence models**	Combination of base models and feature sets
	Keras Neural Networks via TensorFlow [16]
	Convolutional Neural Networks via TensorFlow [16]
	PyPREdictor, a reimplementation of the PREdictor [3]
	Dummy PREdictor as used in [7]
	Wrapper for SVM-MOCCA [7]
	Deep-MOCCA
**Sequence model features**	Operations on feature sets for the definition of models
	Multi-core processing
	Validation
	Prediction threshold calibration
	Genome-wide prediction
	Multi-class model specification via sequence labels
	Retraining

Gnocis provides a flexible and extensible modelling API, with implementations of a variety of models and integrations with Scikit-learn [15] and TensorFlow [16].

### A cross-validation workbench for DNA sequence models with support for imbalanced, multi-class data facilitates unbiased assessment of generalization

An important step when applying machine learning is that of cross-validation, which enables the quantification of model generalization to independent data and the unbiased comparison of models. CRE data is typically imbalanced, with a genome containing relatively few CREs compared to non-CRE regions. Model generalization can be visualized using Receiver Operating Characteristic (ROC) curves and Precision/Recall (PR) curves. PR curves are more informative than ROC curves when data is imbalanced [13]. To our knowledge, no Python cross-validation workbench for imbalanced, multi-class DNA sequence data has been published.

We seized the opportunity to implement a flexible cross-validation workbench for Gnocis that facilitates analyses of model generalization. The modelling API of Gnocis allows the retraining of models. Our cross-validation workbench takes a training set, which can be multi-class, and a binary test set of positives and controls. In order to reflect random variation, Gnocis constructs independent pairs of training and test sets. In order to facilitate multi-class training, when constructing cross-validation training sets, an equal number of examples are randomly selected without replacement from each class, leaving out a desired minimal number of sequences for testing. This procedure yields a balanced training set. When constructing the test sets, sequences are randomly selected from the positives and negatives with a desired ratio, leaving out any sequences that are included in the corresponding training sets. After constructing the cross-validation training and test sets, Gnocis applies models to the sets in order to measure generalization. In order to aid flexible experimentation, a Gnocis cross-validation is constructed as a class that contains pairs of training and test sets, as well as models to cross-validate, and supports the incremental addition of new models. In order to visualize generalization, the Gnocis cross-validation workbench implements the generation of ROC and PR curves with confidence intervals and integrates with Matplotlib [26].

Once models have been trained, prediction thresholds can be set and models can be applied for the genome-wide prediction of CREs. The CREs that are predicted can vary depending on the particular training set that was employed. Gnocis implements cross-validated genome-wide prediction, in which the models trained for each cross-validation repeat are applied. When visualizing measures of predictions, means and confidence intervals are calculated and plotted. When visualizing predicted loci, the fraction of model cross-validation repeats that predict each locus is indicated with prediction opacity.

### Interactive and reproducible analysis

The Python read-eval-print loop (REPL) and Jupyter Notebooks [14] enable the user to interactively write and execute code in Python. Jupyter Notebooks furthermore can store all steps and display formatted tables and graphics. Packages that implement human-readable printout or formatted tables of data structures, such as Pandas [23], enable interactive and reproducible data analysis. Matplotlib [26] can generate figures and display them in Jupyter Notebooks, further empowering Python with Jupyter Notebooks as an analytical platform.

In order to facilitate interactive and reproducible analysis and modelling with Gnocis, we implemented the formatted table output of region sets, sequence sets and extracted feature values. Gnocis implements table objects that can be printed as formatted ASCII text or displayed as formatted HTML tables in Jupyter Notebooks. Additionally, Gnocis tables can be converted to NumPy arrays [22] and Pandas data frames [23]. Gnocis integrates with Matplotlib in order to generate ROC and PR curves for cross-validation and barplots for region overlap statistics. Additionally, Gnocis implements the visualization of genomic regions using Matplotlib.

## Materials and methods

### Genome

We used the *Drosophila melanogaster* genome assembly R5.57 for all analyses, downloaded from FlyBase [32]. We downloaded genes from Ensembl [33] in GTF-format. The genome and the gene annotations are included with Gnocis in the tutorial folder.

### Region sets

For the Kahn et al. PREs [34], we downloaded coordinates from their Supplementary Table S1 and manually converted them to GFF format. For the Enderle et al. PREs [35], we downloaded coordinates from their Supplementary Table 3 and manually converted them to GFF format. We downloaded peaks for the following factors and marks from ModENCODE [6]: Pc (ID: 3957_1816), Psc (ID: 3960_1817), dRING (ID: 5071_1819) and H3K27me3 (ID: 3955_1820).

### Training and cross-validation set

We based the positives in the training set on Kahn et al. PREs [34] (see above). For the unbiased comparison with known PREs at the *invected* and *vestigial* gene loci, we removed PREs that were within 100kb of the bodies of these genes. We extended each PRE from its centre position to a length of 3kb each. Finally, we extracted the underlying genomic sequences (196 sequences).

We generated four sets of non-PREs: dummy genomic sequences (as in [7]), dummy PREs (as in [7]), coding sequences (as in [7]) and genomic non-PREs. For dummy genomic sequences, we trained a 4th-order Markov chain genome-wide and generated 19,600 sequences (100 times as many as there are positives). For dummy PREs, we trained a 4th-order Markov chain on the Kahn et al. PREs [34] and generated 19,600 sequences. For coding sequences, we extracted coding regions from the genome annotation (described above) and merged overlapping regions. We then extracted the genomic sequences, concatenated them and split them into 3kb fragments. For genomic non-PREs, we extracted genomic regions that were depleted of Pc, Psc, dRING and H3K27me3 (see above). We then identified all 3kb windows (with a step size of 250) that were not enriched in any of the four markers, merged the PcG-depleted windows and removed regions within 100kb from the *invected* and *vestigial* gene bodies. Finally, we extracted the sequences from the genome and extracted non-overlapping 3kb windows from the sequences.

### Cross-validation

Cross-validation was performed using functionality implemented in Gnocis, using 20 repeats per model. Genome-wide prediction was performed for each of the 20 repeats of each model, and means and confidence intervals calculated for each measure considered. All confidence intervals were calculated using functionality implemented in Scipy [36, 37], with a t-distribution with 19 degrees of freedom and the confidence level set to 95%.

### PyPREdictor

We re-implemented the PREdictor [3] with Gnocis using a feature network, henceforth called the PyPREdictor. We trained the PyPREdictor with PREs as positives and dummy PREs as negatives (see above), as we did previously in [7]. We used a step size of 250bp and we used the motifs from [3, 7].

### SVM-MOCCA

In our experiments, we used the wrapper for the SVM-MOCCA implementation in the MOCCA suite [38] that is included in Gnocis. We used a window size of 3kb, a step size of 1kb, a quadratic kernel and the motifs from [3, 7]. We trained one SVM-MOCCA model with PREs, dummy genomic sequences, dummy PREs and coding sequences (as in [7]), and one SVM-MOCCA model with PREs, dummy PREs, coding sequences and genomic non-PREs. For the core-PRE prediction, we used the default core prediction algorithm implemented in the MOCCA suite.

### 5-spectrum mismatch SVM

We trained the 5-spectrum mismatch SVM with a quadratic kernel (polynomial degree 2) using a feature network in Gnocis and CUDA SVM, with PREs as positives and genomic non-PREs as negatives. We used a window size of 500bp and a step size of 250bp.

### Convolutional neural network

We constructed a CNN using TensorFlow and Keras, with four convolutional layers, each with 25 three-nucleotide convolutions and followed by an average pooling layer that halved the resolution of the preceding convolution. The final convolution and average pooling layer is followed by a global max pooling layer and a dense softmax layer for class label prediction. We trained the CNN with PREs, dummy PREs, coding sequences and genomic non-PREs. We used a window size of 500bp and a step size of 250bp.

### Deep-MOCCA

**Fig 1:**
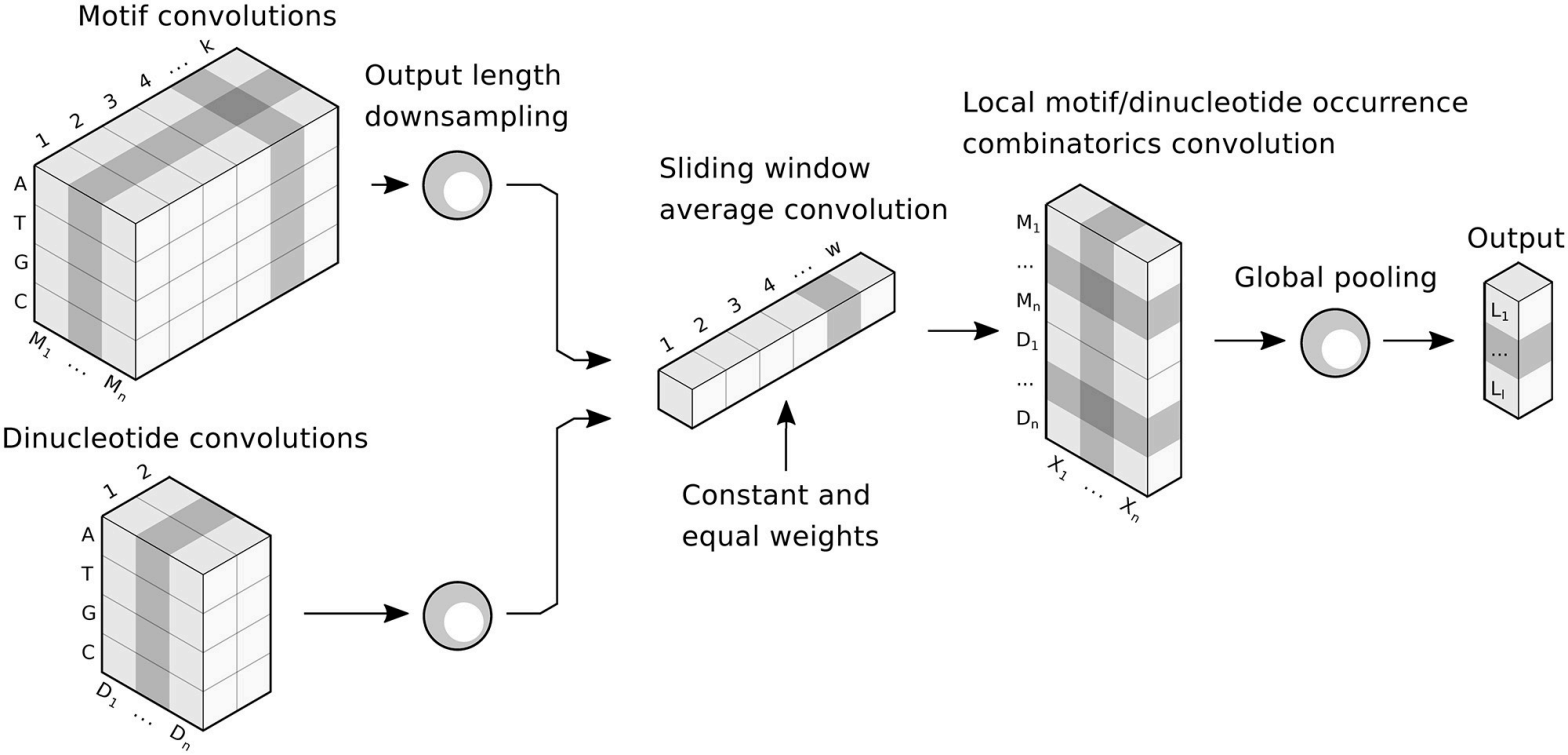
Deep-MOCCA schematic. Deep-MOCCA is a convolutional neural network architecture that mimics the structure of SVM-MOCCA [7].

Deep-MOCCA uses a layer of sequence convolutions and dinucleotide convolutions. For efficiency, the input convolutions are followed by an average pooling layer for 10-fold downscaling of resolution. In order to model local motif occurrence combinatorics within a bidirectional cut-off distance, the third layer is a convolution of length 50 (corresponding to 500bp) with constant and equal weights (weight = 1/50), effectively averaging with a sliding window. This is followed by a layer of motif/dinucleotide pairing convolutions of width 1. Finally, a global max pooling layer and a dense softmax layer are used to predict sequence labels. The model architecture is visualized in Fig 1.

In our experiments, we used 25 motif convolutions of length 10, 25 dinucleotide convolutions and 25 pairing convolutions. We used a window size of 500bp and a step size of 250bp. We trained Deep-MOCCA with PREs, dummy PREs, coding sequences and genomic non-PREs for 350 epochs.

### Software and packages

The present analyses were performed using Python version 3.8.5 and Gnocis version 0.9.12. For Support Vector Machines, we used the implementation available in Scikit-learn [15] version 0.23.2. For neural networks, we used the TensorFlow [16] version 2.4.1 package for Python. For CuPy, we used version 7.8.0. For the calculation of confidence intervals we used Scipy [36, 37] version 1.6.3. For SVM-MOCCA, we used the implementation in the MOCCA suite [38] version 1.4.7.

## Results

We applied Gnocis to the problem of modelling PREs. The code used to generate all results is available in a Jupyter Notebook on GitHub, at https://github.com/bjornbredesen/gnocis/tree/master/tutorial/tutorial.ipynb.

### A quadratic 5-spectrum mismatch SVM achieves moderate generalization to independent PREs without prior motif knowledge

We have previously found that SVM-MOCCA improves the generalization to independent PREs over the PREdictor [7]. Gnocis implements the k-spectrum kernel, which has previously been applied with SVMs for the prediction of Polycomb targets in *Xenopus tropicalis* [29] and for other regulatory elements [28, 39]. An important benefit of the k-spectrum kernel is that no prior motif knowledge is required. Gnocis additionally implements the k-spectrum mismatch kernel [40], which to our knowledge has not previously been applied to PREs. We were interested in how well a k-spectrum mismatch SVM would generalize to PREs.

We cross-validated the PyPREdictor (re-implementation of the PREdictor [3]), SVM-MOCCA and a 5-spectrum mismatch SVM with PREs and non-PREs (see Materials and methods). For the PyPREdictor, we used PREs as positives and dummy PREs as negatives (as in [7]). For the 5-spectrum mismatch SVM, we reasoned that the dummy PREs, which model 5-mer occurrence frequencies of PREs, are too similar in the model feature space. For increased realism, we trained the 5-spectrum mismatch SVM with PREs as positives and genomic non-PREs (see Materials and methods) as negatives. For SVM-MOCCA, we trained one model with PREs as positives and dummy genomic, dummy PREs and coding sequences as negatives, as in [7]. Additionally, we trained an SVM-MOCCA model where we replaced the dummy genomic sequences with genomic non-PREs (SVM-MOCCA T2021). For the k-spectrum mismatch SVM, we used a quadratic (second-degree polynomial) kernel in order to model motif pairing—which is predictive of PREs [3]—, and we set k to 5, since multiple known PRE motifs are 4-mers or 5-mers (GTGT, GCCAT and GAGAG).

**Fig 2:**
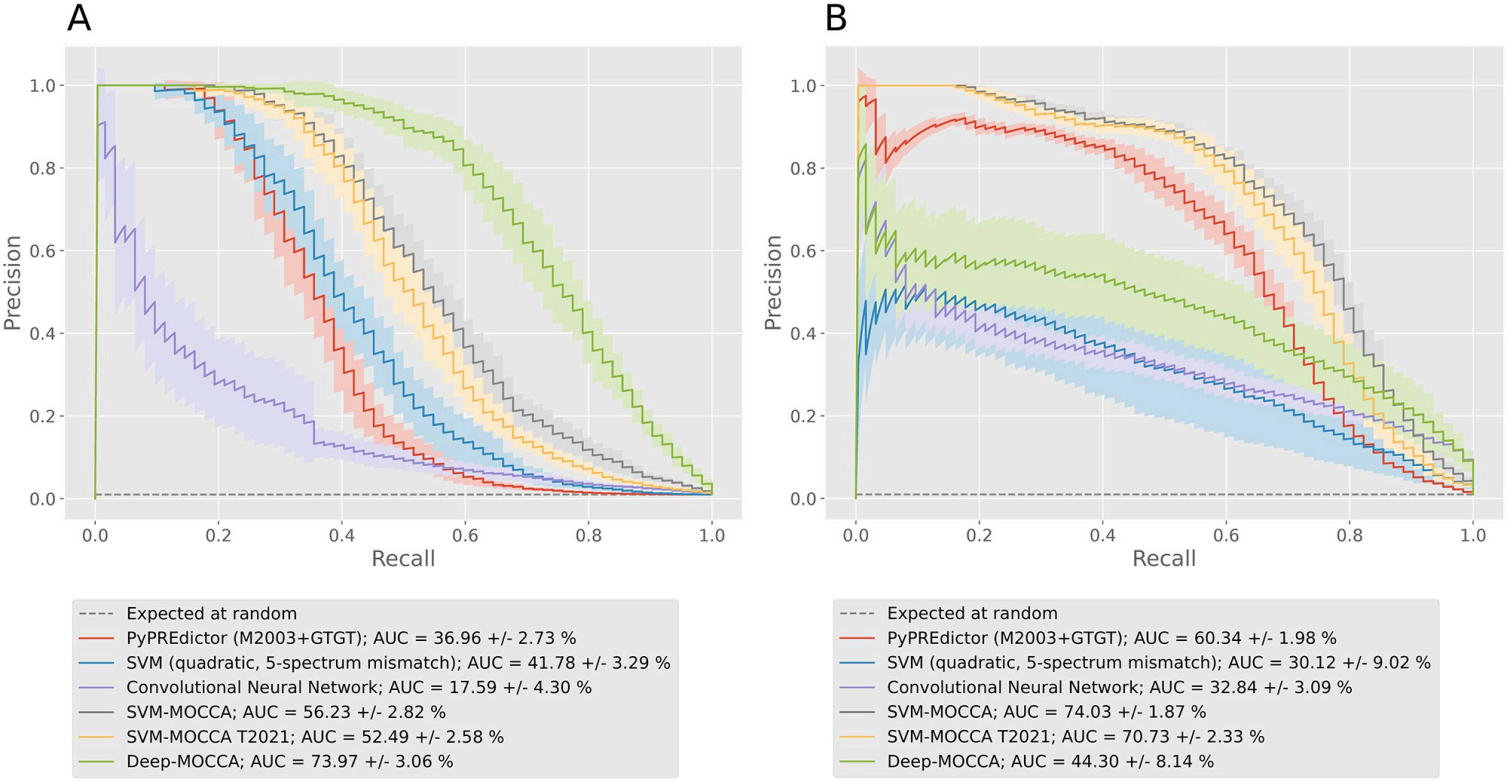
Cross-validation Precision/Recall curves. We cross-validated our models trained with PREs and non-PREs, and tested with independent A) PREs versus dummy PREs and B) PREs versus coding sequences.

For PREs versus dummy PREs, the quadratic 5-spectrum mismatch SVM achieves a 1.13-fold improvement in PRC AUC over that of the PyPREdictor (Fig 2, panel A). Both SVM-MOCCA models yield similar and superior generalization over that of the spectrum SVM (Fig 2, panel A). For PREs versus coding sequences (Fig 2, panel B), the PyPREdictor and SVM-MOCCA achieve high generalization (PRC AUC >60%), and the 5-spectrum mismatch SVM achieves moderate generalization (PRC AUC >30%). We also trained the quadratic 5-spectrum mismatch SVM with PREs and dummy PREs, which resulted in overfitting to the negative training set and close to random generalization with other negative test sets (S1 Fig).

In conclusion, the quadratic 5-spectrum mismatch SVM achieves moderate generalization to independent PREs, without prior motif knowledge. The moderate generalization to PREs versus dummy PREs indicates that the spectrum SVM learns to model motif pair occurrence frequencies. Overall, the quadratic 5-spectrum mismatch SVM achieves respectable generalization to independent PREs without prior motif knowledge. However, SVM-MOCCA—which uses known motifs—yields superior generalization.

### GPU-based SVM application reduces running time by an order of magnitude

Gnocis implements support for multiprocessing for machine learning models. Additionally, for SVMs, Gnocis implements GPU-based model application. We were interested in how the parallelism implemented in Gnocis affects run-time performance.

In order to yield a fair comparison, we trained three 5-spectrum mismatch kernel SVMs on the same data (PREs as positives and genomic non-PREs as negatives) and the same hyper-parameters (quadratic kernel): one with multiprocessing disabled, one with multiprocessing enabled (12 processes) and one with GPU-based application. We timed the application of each SVM to the same set of 19,600 dummy genomic sequences. The run-times are listed in Table 4.

**Table 4:** Multiprocessing and GPU application of SVMs significantly reduces run-times.

Application method	Running time (h:mm:ss)
1 core	0:10:24
12 cores/threads	0:05:18
GPU	0:01:28

We applied a quadratic 5-spectrum mismatch SVM to 19,600 3kb-long dummy genomic sequences using a single core, twelve cores/threads and a GPU. CPU: Intel Core i9–9900K, 3.6 GHz, 8 cores, 16 threads. GPU: GeForce GTX 980.

Multiprocessing almost halves the running time. GPU-based model application further improves running time, shortening it to almost a tenth of the single-threaded model application.

In conclusion, parallel application of SVMs significantly reduces run-time cost. That multithreaded run-time cost does not scale with the number of threads can be attributed to how Python implements multiprocessing. GPU-based application of SVMs further reduces running time by an order of magnitude.

### Convolutional neural networks achieve low to moderate generalization to PREs

Convolutional neural networks (CNNs) [41] have recently shown great success in computer vision [12]. A successful CNN architecture in computer vision is one with multiple layers of small convolutions, combined by pooling layers [12]. A convolution over one-hot-encoded DNA sequences is effectively a Position Weight Matrix (PWM). CNNs have not previously been applied to the task of modelling PREs, and we were interested in how well CNNs would perform in this modelling task.

We trained a CNN with four layers of 25 3bp convolutions each (see Materials and methods), and a dense softmax layer with four classes: PREs, dummy PREs, coding sequences and genomic non-PREs.

The CNN achieved low generalization (PRC AUC <20%) to PREs versus dummy PREs (Fig 2, panel A), and moderate generalization (PRC AUC >30%) to PREs versus coding sequences (Fig 2, panel B).

In summary, a multilayer CNN with short convolutions achieves low to moderate generalization to independent PREs. The low generalization might be attributed to multiple factors. The CNN preserves positional information, which previous models do not, and which increases model complexity but may be irrelevant to the modelling problem. It is also possible that tuning the number of layers, the number of convolutions per layer and the length of the convolutions may improve generalization.

### The convolutional neural network architecture Deep-MOCCA improves the state-of-the-art of PRE models without prior motif knowledge

There is significant freedom in how artificial neural networks can be architected. Inspired by this freedom and the high generalization of SVM-MOCCA to independent PREs [7], we were interested in how an artificial neural network architecture with similar model structure would perform at the task of modelling PREs.

We designed Deep-MOCCA, a convolutional neural network architecture that mimics the structure of SVM-MOCCA by modelling local motif occurrence combinatorics and dinucleotide patterns but without the need for prior motif knowledge. The model architecture is described in detail in Materials and methods. In order to learn motifs, Deep-MOCCA has a layer of longer convolutions, and in order to model dinucleotides, a layer of 2bp convolutions. These two convolutional layers are concatenated. In order to model local motif occurrence combinatorics, Deep-MOCCA uses a sliding window averaging layer and a layer of single-nucleotide pairing convolutions. Finally, Deep-MOCCA outputs predicted class probabilities with a dense softmax layer. We trained Deep-MOCCA with four classes: PREs, dummy PREs, coding sequences and genomic non-PREs.

Deep-MOCCA achieves the highest generalization to PREs versus dummy PREs of all models tested, with a 1.31-fold improvement in PRC AUC over that of SVM-MOCCA (Fig 2, panel A). For PREs versus coding sequences, Deep-MOCCA achieves a lower generalization than that of SVM-MOCCA, but a higher one than the 5-spectrum mismatch SVM and the conventional CNN (1.35-fold improvement in PRC AUC; Fig 2, panel B).

In summary, we have developed a convolutional neural network architecture, Deep-MOCCA, that improves the state-of-the art for PRE models that require no prior motif knowledge by exploiting prior knowledge about successful PRE sequence model structure (SVM-MOCCA [7]). Deep-MOCCA significantly improves generalization over a more conventional CNN with more layers and smaller convolutions. Part of the improvement in Deep-MOCCA over the conventional CNN may be attributed to a lower model complexity (Deep-MOCCA has 2,629 trainable parameters, the conventional CNN has 6,129), which in turn may reduce overfitting. Also, Deep-MOCCA discards spatial information beyond local pairing. When motifs are known, there is still a benefit in including these motifs in PRE models, as SVM-MOCCA achieves superior generalization to PREs versus coding sequences. Gnocis includes an implementation of Deep-MOCCA that can be adapted to new modelling problems.

### Deep-MOCCA precisely predicts independent PREs without prior motif knowledge

We were interested in how well models implemented in Gnocis can predict PREs genome-wide.

We calibrated the prediction threshold of each model (six models with 20 cross-validation repeats each) for an expected genome-wide precision of 80%, based on independent PREs from the corresponding cross-validation test set and a 7th-order Markov chain trained genome-wide. We then applied each model genome-wide for prediction of candidate PREs using a sliding window, predicting windows with scores above the prediction threshold and merging overlapping predictions. For SVM-MOCCA, we additionally predicted core-PREs using the algorithm from the MOCCA suite [38]. For validation of predictions, we did not train on regions from the *invected* and *vestigial* loci, where multiple known PREs reside. Additionally, we extracted the subset of PREs from Enderle et al. (2011) [35] that are at least 1kb away from all PREs from the Kahn et al. (2014) [34] set.

**Fig 3:**
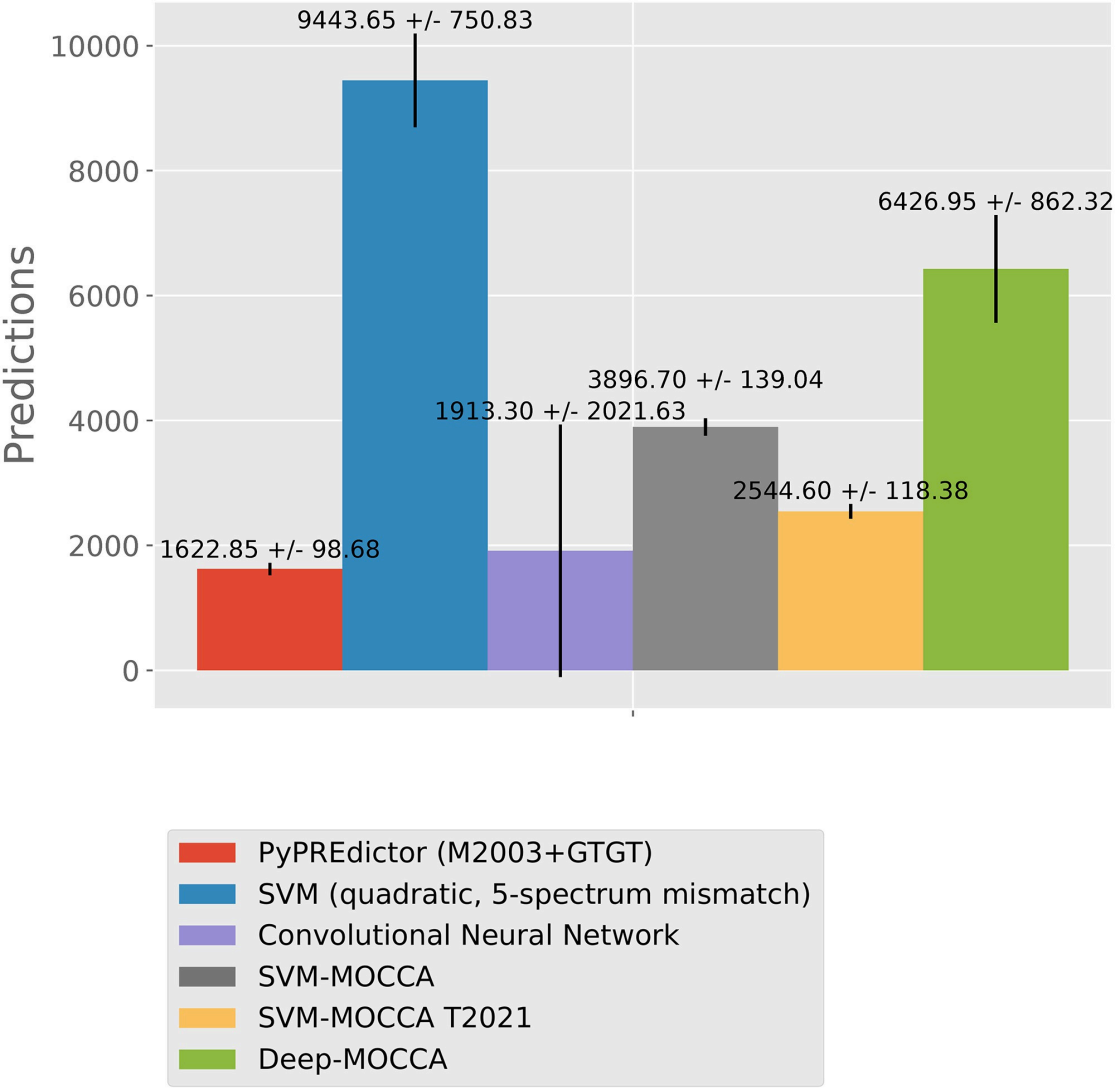
Numbers of predictions.

**Fig 4:**
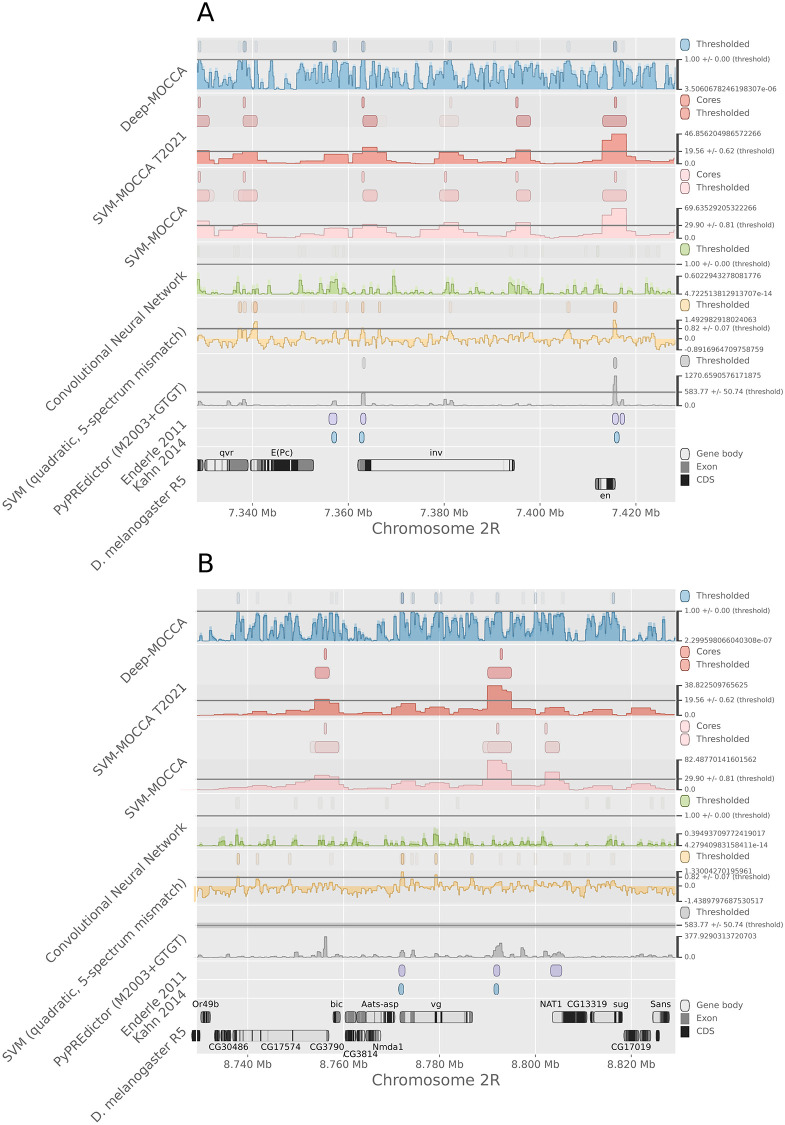
Predictions at the A) *invected* and B) *vestigial* loci. Visualized using the Gnocis genomic track plotting, which uses Matplotlib [26]. Opaque predictions are predicted in the majority of cross-validation repeats, and semi-transparent predictions in a subset of repeats.

**Fig 5:**
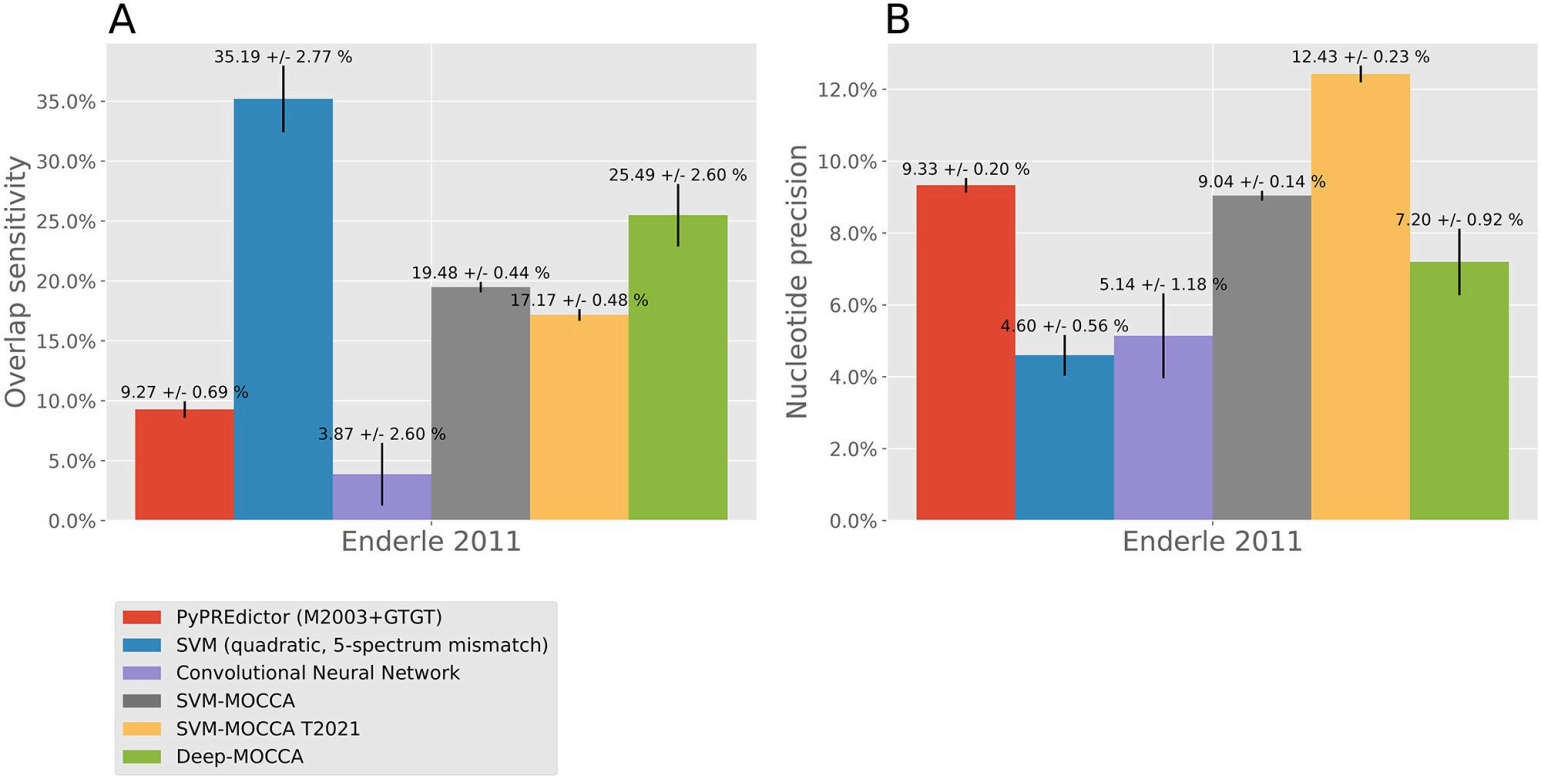
Prediction overlap with experimental data. A) Overlap sensitivity of predictions to Enderle et al. (2011) [35] PREs. B) Nucleotide precision of predictions to Enderle et al. (2011) [35] PREs. In order to avoid bias, for the calculations in both A) and B), we removed PREs from [35] and predictions that were within 1kb of overlapping with a Kahn et al. (2014) [34] PRE.

Of the models tested, the quadratic 5-spectrum mismatch SVM yielded the largest number of candidate PRE predictions genome-wide and Deep-MOCCA the second largest (Fig 3). Training the SVM-MOCCA model with genomic non-PREs instead of dummy genomic sequences resulted in a drop of the number of predictions. At the *invected* locus, Deep-MOCCA predicts three known PREs (Fig 4, panel A). At the *vestigial* locus, Deep-MOCCA predicts one known PRE for the majority of cross-validation repeats, and another known PRE for a subset of repeats (Fig 4, panel B). In addition, Deep-MOCCA predicts multiple other regions for subsets of repeats. At the *invected* locus, the 5-spectrum SVM and both SVM-MOCCA models predict two out of three known PREs (Fig 4, panel A). At the *vestigial* locus, the 5-spectrum SVM predicts one known PRE, both SVM-MOCCA models predict one (different) known PRE, and the SVM-MOCCA model trained with dummy genomic sequences predicts an additional PRE (Fig 4, panel B). The conventional CNN predicts no known PREs for either of the two loci. Of the models tested, the quadratic 5-spectrum mismatch SVM achieves the highest sensitivity to independent PREs from [35], with Deep-MOCCA in second and SVM-MOCCA in third (Fig 5). SVM-MOCCA trained with genomic non-PREs achieves the highest nucleotide precision (fraction of predicted nucleotides that land inside a [35] PRE), with the PyPREdictor in second place, SVM-MOCCA trained with dummy genomic sequences in third and Deep-MOCCA in fourth.

In conclusion, Deep-MOCCA precisely predicts independent PREs without prior motif knowledge. Of the models tested without prior motif knowledge, Deep-MOCCA achieves the second highest sensitivity and the highest nucleotide precision. Nucleotide precision is low for all models (<13%), which is expected if experimental signals of PcG-binding may be shifted from the PREs. Furthermore, our models may predict PREs that are not active in the cells that Enderle et al. (2011) [35] used.

## Discussion

Gnocis is a versatile and extensible system for interactive and reproducible analysis and modelling of CRE sequences, and for predicting candidate CREs genome-wide. Gnocis fills a gap left by existing Python packages by implementing the base functionality that is necessary in order to efficiently combine machine learning methods and feature sets. Gnocis provides data preparation facilities and feature-rich APIs for feature set and model specification and application. The data preparation facilities implement common data preparation operations and employ standardized file formats, streamlining the use of published data, integration with external tools and collaboration. In addition to being useful for the preparation and handling of data for CRE machine learning, the data handling facilities in Gnocis can also be useful for general DNA sequence bioinformatics.

The Gnocis feature set API provides the user with a flexible vocabulary for the specification and application of sequence feature sets, with integration with NumPy [22] and Pandas [23] for advanced analyses. In order to enable efficient feature extraction, feature sets in Gnocis are implemented as graphs that can be transformed via a variety of operations. This enables the user to specify feature sets and models with a short syntax, and simplifies retraining, for example for cross-validation. To our knowledge, Gnocis is the first DNA sequence feature package for Python to employ this design. The modelling API implements common procedures for model validation and prediction. For additional efficiency, the modelling API implements multiprocessing support.

When multiple candidate models are available, it is useful to have a platform for unbiased benchmarking. Gnocis provides a cross-validation engine that constructs multiple training and test sets, trains and applies models, and calculates measures of generalization. The Gnocis cross-validation engine supports imbalanced, multi-class data.

In order to facilitate interactive data analysis and modelling, Gnocis integrates with IPython [21] and Matplotlib [26]. Gnocis outputs tables for sequence feature enrichment, simplifying interactive analysis. Gnocis also implements visualization of model generalization via Receiver Operating Characteristic (ROC) and Precision/Recall curves, with means and confidence intervals visualized for cross-validation. In order to probe predictions at genomic loci of interest, Gnocis implements visualization of genomic tracks, enabling visual inspection of genomic loci in Jupyter Notebooks [14].

To demonstrate the utility and ease of use of Gnocis, we applied six models for the prediction of PREs: the PyPREdictor (a Python re-implementation of the PREdictor method [3]), a quadratic 5-spectrum mismatch SVM, a (conventional) CNN, two versions of SVM-MOCCA, and Deep-MOCCA—a neural network architecture inspired by SVM-MOCCA. The 5-spectrum SVM achieves the highest sensitivity to independent PREs, but also the lowest precision. Deep-MOCCA achieves the second highest sensitivity to independent PREs and the highest precision of models without prior motif knowledge. SVM-MOCCA achieves the highest precision of the models tested. Our present work is the first to apply Convolutional Neural Networks to the modelling of PRE sequences. Notably, there are numerous potential network structures that can be employed for a CNN, and other network architectures may outperform the ones we tested here. Gnocis provides the user with the tools necessary in order to test new neural network architectures. Additionally, Gnocis includes Deep-MOCCA, which can be trained on new problems. We previously demonstrated the applicability of SVM-MOCCA to new problems by training it to predict Boundary Elements [38]. We expect similar broader applicability for Deep-MOCCA, and as Deep-MOCCA requires no prior motif knowledge, it may also be interesting to apply it to problems where motif knowledge is lacking. Gnocis is species agnostic and our methods can be trained for prediction tasks in other species where appropriate data can be collected. For example, Support Vector Machines have previously been applied for modelling H3K27me3 nucleation sites in Western clawed frog (*X. tropicalis*) [29], and methods implemented in Gnocis could in principle be trained using the same or similar data.

The PyPI package manager makes Gnocis easy to install on multiple operating systems and, with Gnocis having no dependencies, further improves the portability of our package. In addition to internally implementing a broad suite for data preparation, Gnocis abstractly implements DNA sequence feature spaces and sequence modelling, facilitating the exploration of different modelling approaches, both in terms of feature space definitions and of machine learning methods. The suite of tools that Gnocis provides can aid in elucidating the sequence criteria that define a CRE class, and in predicting new CREs genome-wide.

## Software availability and requirements

- Project name: Gnocis
- Project home page: https://github.com/bjornbredesen/gnocis
- Operating systems: GNU/Linux, Windows, MacOS X
- Programming languages: Python, Cython
- Requirements: Python 3.6/3.7/3.8/3.9
- License: MIT license

The code for generating all results presented here is available as Jupyter Notebooks on the Gnocis GitHub repository.

## Supporting information

S1 Fig Training with dummy PREs as negatives leads to overfitting to the training classes. Shown are the dummy PREdictor, the PyPREdictor trained with PREs (positives) and dummy PREs (negatives), a quadratic 5-spectrum mismatch kernel SVM trained with PREs (positives) and genomic non-PREs (negatives) and finally a quadratic 5-spectrum mismatch kernel SVM trained with PREs (positives) and dummy PREs (negatives). Models were tested with A) PREs versus dummy PREs, B) PREs versus coding sequences and C) PREs versus genomic non-PREs. AUC is high for the SVM trained with dummy PREs when tested with dummy PREs (A) but low otherwise (B, C).
(PDF)
