# Supplementary material for: Gnocis: An integrated system for interactive and reproducible analysis and modelling of cis-regulatory elements in Python 3
Source: PLoS One. 2022 Sep 9;17(9):e0274338. doi: 10.1371/journal.pone.0274338 (PMC9462789; doi:10.1371/journal.pone.0274338)
Supplement: S1 Fig — Shown are the dummy PREdictor, the PyPREdictor trained with PREs (positives) and dummy PREs (negatives), a quadratic 5-spectrum mismatch kernel SVM trained with PREs (positives) and genomic non-PREs (negatives) and finally a quadratic 5-spectrum mismatch kernel SVM trained with PREs (positives) and dummy PREs (negatives). Models were tested with A) PREs versus dummy PREs, B) PREs versus coding sequences and C) PREs versus genomic non-PREs. AUC is high for the SVM trained with dummy PREs when tested with dummy PREs (A) but low otherwise (B, C). (PDF) [file pone.0274338.s001.pdf]

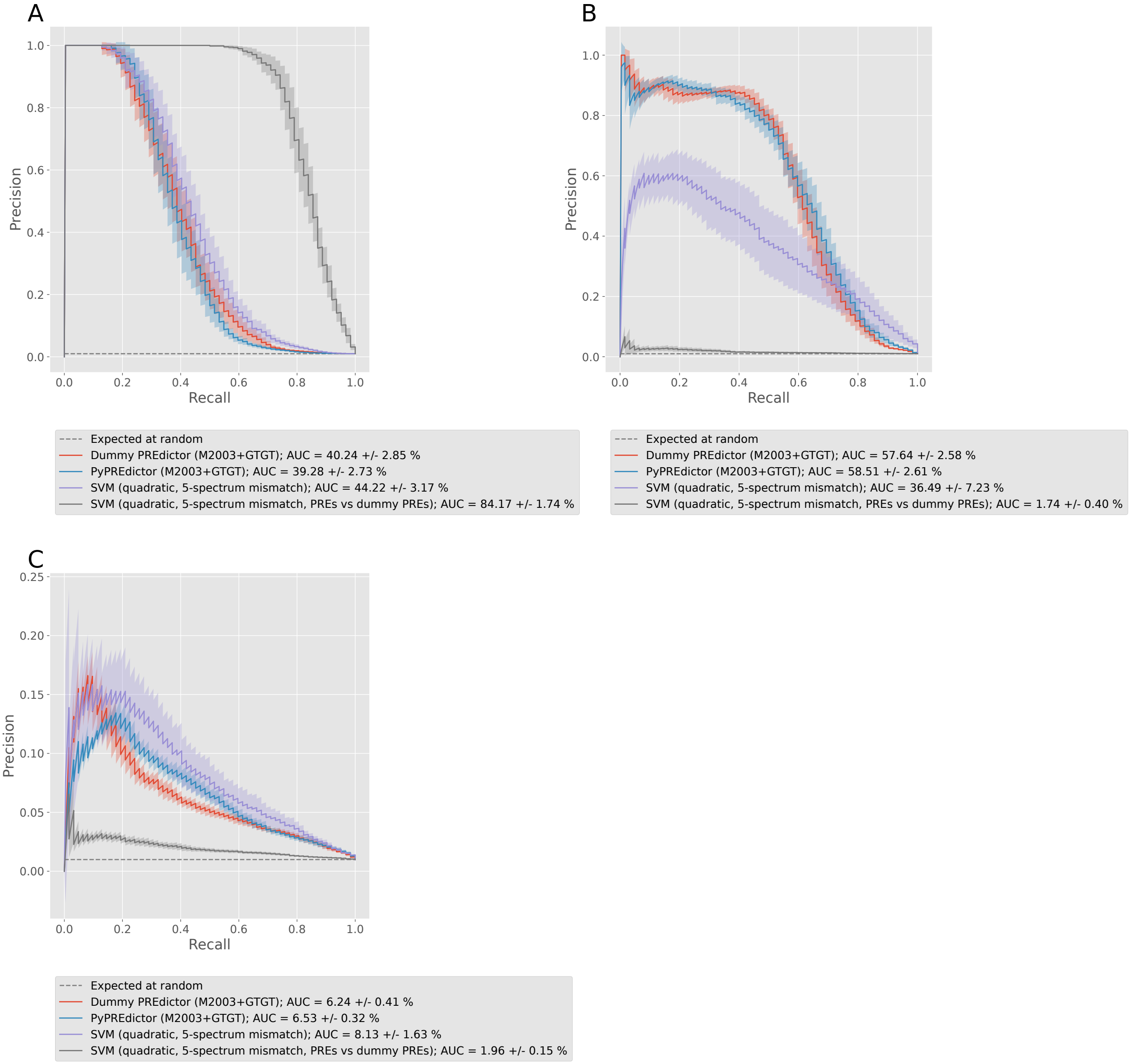

S1 Fig. Training with dummy PREs as negatives leads to overfitting to the training classes. Shown are the dummy PREdictor, the PyPREdictor trained with PREs (positives) and dummy PREs (negatives), a quadratic 5-spectrum mismatch kernel SVM trained with PREs (positives) and genomic non-PREs (negatives) and finally a quadratic 5-spectrum mismatch kernel SVM trained with PREs (positives) and dummy PREs (negatives). Models were tested with A) PREs versus dummy PREs, B) PREs versus coding sequences and C) PREs versus genomic non-PREs. AUC is high for the SVM trained with dummy PREs when tested with dummy PREs (A) but low otherwise (B, C).
